# Transcriptome Analysis of Pepper (*Capsicum annuum*) Revealed a Role of 24-Epibrassinolide in Response to Chilling

**DOI:** 10.3389/fpls.2016.01281

**Published:** 2016-08-29

**Authors:** Jie Li, Ping Yang, Jungen Kang, Yantai Gan, Jihua Yu, Alejandro Calderón-Urrea, Jian Lyu, Guobin Zhang, Zhi Feng, Jianming Xie

**Affiliations:** ^1^Department of Facility Horticulture Science, College of Horticulture, Gansu Agricultural UniversityLanzhou, China; ^2^Department of Crop Cultivation and Farming System, College of Agronomy, Gansu Agricultural UniversityLanzhou, China; ^3^Department of Vegetable Genetics and Breeding, Beijing Vegetable Research Center, Beijing Academy of Agriculture and Forestry SciencesBeijing, China; ^4^Semiarid Prairie Agricultural Research Centre, Agriculture and Agri-Food CanadaSwift Current, SK, Canada; ^5^Gansu Provincial Key Lab of Aridland Crop Science, Gansu Agricultural UniversityLanzhou, China; ^6^Department of Biology, California State University FresnoFresno, CA, USA

**Keywords:** pepper, Brassinosteroid, chill-stress, transcriptome, RNA sequencing

## Abstract

Brassinosteroids (BRs) have positive effects on many processes during plant growth, development, and various abiotic stress responses. However, little information is available regarding the global gene expression of BRs in response to chilling stress in pepper. In this study, we used RNA sequencing to determine the molecular roles of 24-epibrassinolide (EBR) during a chilling stress response. There were 39,829 transcripts, and, among them, 656 were differently-expressed genes (DEGs) following EBR treatment (Chill+EBR) compared with the control (Chill only), including 335 up-regulated and 321 down-regulated DEGs. We selected 20 genes out of the 656 DEGs for RT-qPCR analysis to confirm the RNA-Seq. Based on GO enrich and KEGG pathway analysis, we found that photosynthesis was significantly up-enriched in biological processes, accompanied by significant increases in the net photosynthetic rate (Pn), Fv/Fm, and chlorophyll content. Furthermore, the results indicate that EBR enhanced endogenous levels of salicylic acid (SA) and jasmonic acid (JA) while suppressing the ethylene (ETH) biosynthesis pathway, suggesting that BRs function via a synergistic cross-talk with SA, JA, and ETH signaling pathways in response to chilling stress. In addition, EBR induced cellulose synthase-like protein and UDP-glycosyltransferase, suggesting a contribution to the formation of cell wall and hormone metabolism. EBR also triggered the calcium signaling transduction in cytoplasm, and activated the expression of cellular redox homeostasis related genes, such as *GSTX1, PER72*, and *CAT2*. This work, therefor, identified the specific genes showed different expression patterns in EBR-treated pepper and associated with the processes of hormone metabolism, redox, signaling, transcription, and defense. Our study provides the first evidence of the potent roles of BRs, at the transcription level, to induce the tolerance to chilling stress in pepper as a function of the combination of the transcriptional activities, signaling transduction, and metabolic homeostasis.

## Introduction

Chilling stress affects the geographical distribution of many important vegetables such as pepper (*Capsicum annuum* L.; Sanghera et al., 2011). This abiotic stress adversely affects plant growth and development and the yield and quality of many crops (Janská et al., 2010; Ren et al., 2014). In Northern China, where greenhouse cultivation is used for vegetable production, it is very common for plants suffer a chilling injury during the fall-to-winter transition period when a sudden temperature drops often leads to early frost damage. In vegetables, low temperatures lead to the arrest of growth or abortion of flower buds, resulting in significant yield and economic losses (Chinnusamy et al., 2007).

Plants have evolved pleiotropic and intricate regulatory functions to defend against environmental stresses (Xia et al., 2009). Under stress, plants produce a number of phytohormones, such as salicylic acid (SA), brassinosteroids (BRs), and abscisic acid (ABA), which play a critical role in the perception of external signals and the activation of defense mechanism within plants. Brassinosteroids, a group of naturally occurring plant steroids, have been shown to provide positive effects on the regulation of plant growth and a broad spectrum of physiological responses to abiotic stresses, such as high and low temperature stress (Mazorra et al., 2002; Bajguz, 2009), drought (Yuan et al., 2010), and salinity injury (Liu et al., 2014). For example, BRs increase thermotolerance of plants by inducing heat shock protein synthesis and gene expression for heat tolerance (Dhaubhadel et al., 2002; Dhaubhadel and Krishna, 2008). BRs induce cold-related gene expression in *Brassica napus* and *Arabidopsis thaliana* (Kagale et al., 2007). Ubiquitin-conjugating enzyme (*UBC32*) as a critical gene involved in BRI1 biosynthesis and ER-associated protein degradation (*ERAD*) pathway positively regulates BR-induced salt tolerance (Cui et al., 2011). A synergistic interaction among BR signaling and the production of reactive oxygen species (ROS) induces the gene expression of respiratory burst oxidase homolog (RBOH), whereas encoding NADPH oxidase and NADPH oxidase trigger apoplastic ROS accumulation, activating MAPKs to increase plant tolerance to stress (Hao et al., 2013).

RNA sequencing techniques have been used to investigate global expression profiles and reveal the signal transduction pathways involved in the resistance network under various stresses (Liu et al., 2015; Wang J. et al., 2015). The genome of pepper has been recently sequenced (Qin et al., 2014), which provides a valuable resource for molecular-based investigations for stress tolerance in plants. In previous studies, we found that exogenous BRs alleviated low temperature stress in pepper by enhancing antioxidant capacity and maintenance of photosystem II (Li et al., 2015a,b). However, it is unknown regarding the specific gene expression profile of BRs-induced chilling tolerance in pepper, and the genomic characteristics of the BRs-induced tolerance were undefined.

Here, we reveal the genes associated with chilling stress and the associated signaling pathways mediated by BRs using RNA-seq analysis. The goals of the present study were to (i) provide insights into the pepper leaf transcriptome response to BRs under chilling stress; and (ii) uncover the genes and pathways that are associated with BRs-induced stress tolerance in pepper.

## Materials and methods

### Plant material and stress treatment

Pepper seeds (cv. “Xiangyan NO.16”) were germinated in the dark for 72 h at 28°C before being transplanted into plastic pots containing a mixture of vermiculite and peat (1:2, v: v) for subsequent growth. The seedlings were grown in an intelligent greenhouse with 25/15°C (day/night), photon flux density of 350–400 μmol m^−2^ s^−1^, 12-h photoperiod, and relative humidity of 60–80%.

We used six different concentrations (0, 10, 1, 0.1, 0.01, and 0.001 μM) to manipulate BRs levels in pepper plants as described by Li et al. (2015b). We used 0.1 μM EBR as an optimum EBR concentration based on the results of the prior experiment where various concentrations were studied and the optimum level was identified.

At the 6–7 leaf stage (50 days after planting), the seedlings were divided into the following two groups: one was sprayed with 0.1 μM EBR solution, and the other was sprayed with the same volume of double distilled water. Twenty-four hours later, seedlings in both groups were transferred to a controlled growth chamber with temperature at 15/5°C (day/night), photon flux density at 100 μmol m^−2^ s^−1^, 12-h photoperiod, and relative humidity of 80%. Each treatment was replicated three times at the growth chamber, and each replicate had 20 plants. At day 7 of chilling, we collected two biological replicates of each treatment for sequencing, and each biological replicate had three plants. All samples were collected at the same time, ground into powder in liquid nitrogen and stored at −80°C for further use.

These samples were labeled Chill+EBR (EBR treatment under chilling stress) and Chill (control).

### RNA extraction and RNA-seq

Total RNA was extracted from the Chill and Chill+EBR samples (leaves) with Trizol reagent (Invitrogen, Carlsbad, CA, USA) using method described by Hu et al. (2012). The total RNA extraction was divided into two aliquots; one aliquote was used for RNA-sequencing, and the other was used for real-time PCR. The RNA-sequencing was performed using an Illumina HiSeq 4000 platform (Illumina, San Diego, CA, USA) at Novogene Bioinformatics Technology Co., China. Each sample generated more than 6 gigabyte of data. The clean reads were filtered from raw sequencing data and the low-quality reads containing unknown nucleotides or adaptor sequences were removed; this procedure was performed in accordance with the method of Chen et al. (2014). The filtered clean reads were aligned to *C. annuum* reference genome (http://peppersequence.genomics.cn/page/species/download.jsp).

### Differential expression analysis

We performed differential expression analysis for both Chill and Chill+EBR treatments based on the DESeq R package, which allowed for statistical analysis using the negative binomial distribution model (Wang et al., 2010). To control the false discovery rate, we adjusted the resulting *p*-values according to Benjamini and Hochberg's approach (Benjamini and Hochberg, 1995), where an adjusted *p* < 0.05 is accepted to represent differentially expressed genes (DEGs).

We performed gene ontology (GO) enrichment analysis of the DEGs according to the GOseq R package, and GO terms with *q* < 0.05 were regarded as significantly enriched (Young et al., 2010). We carried out the statistical enrichment of the differential expression genes in Kyoto Encyclopedia of Genes and Genomes (KEGG) pathways using KOBAS software (Xie et al., 2011).

### Validation of DEGs by real-time quantitative PCR

Twenty transcript genes were selected for the qRT-PCR assay; the genes and gene-specific primers used are summarized in Table S1. *Actin* was used as an internal reference. qRT-PCR was performed using SYBR-Green (ABI-Invitrogen, California, USA) on an ABI 7900 Fast Real-Time PCR Detection System (Applied Biosystems, Carlsbad, USA). A real-time RT-PCR reaction (20 μl) included 10 μl of 2 × SuperReal PreMix Plus, 2 μl cDNA, 1 μl of each primer and 6 μl ddH_2_O, and it proceeded for 40 cycles. The relative expression levels of the selected 20 genes normalized to the expression level of *actin* (internal reference control) were calculated from cycle threshold values using the 2^−ΔΔCt^ method (Livak and Schmittgen, 2001).

### Chlorophyll content and net photosynthetic rate

The chlorophyll a and b concentrations were determined according to the method of Arnon (1949). Net photosynthetic rate (Pn) in the fully expanded leaves of pepper plants was measured by Ciras-2 portable photosynthesis system (PP Systems, USA).

### Chlorophyll fluorescence determination

The chlorophyll fluorescence parameters in pepper leaves were measured using a pulse-modulated fluorometer (FMS-2, Hansatech, Norfolk, UK). Pepper seedlings were placed in the dark for 30 min and were prepared for the determination of the Fv/Fm.

### Analysis of hormones and hormone metabolites

Pepper leaf tissues treated with DDH_2_O or 0.1 μM EBR under chilling stress were collected in liquid nitrogen and stored at −80°C. For analysis of IAA and ETH metabolites, SA and JA, leaf tissues were lyophilized using a freeze dryer; these procedures were in accordance with the methods of published research (Chiwocha et al., 2003; Peng and Zhou, 2009). Indole acetic acid oxidase (IAAO) activity was measured on acetone extract leaves by measuring residual IAA following incubation and agitation in the dark at 30°C. One unit of IAAO activity was expressed as 1 mg of IAA destroyed per milligram of protein per minute. ACC content and ACC synthase (ACS) activity was determined according to the method of Prasad and Cline (Prasad and Cline, 1987).

## Results

### Mapping and quantitative assessment of iiiumina sequence

We constructed two libraries from Chill and Chill+EBR for RNA-Seq. A total of 32.49 million (Chill) and 39.86 million (Chill+EBR) reads were generated. After removing low-quality regions, adapters, and possible contamination, we obtained more than 3 giga base clean bases with a Q20 percentage over 92%, Q30 percentage over 86%, and a GC percentage between 42.9 and 43.7% (Table 1).

**Table 1:** **Summary of sequence assembly after illumina sequencing**.

**Sample name^a^**	**Raw reads**	**Clean reads**	**Clean bases Gb**	**Error rate (%)**	**Q20^b^(%)**	**Q30^c^(%)**	**GC content^d^(%)**
Chill1-1	33,040,705	32,108,541	4.01	0.04	94.63	89.54	42.91
Chill1-2	33,040,705	32,108,541	4.01	0.04	93.08	87.4	42.92
Chill2-1	31,938,987	30,969,153	3.87	0.04	94.44	89.22	43.38
Chill2-2	31,938,987	30,969,153	3.87	0.04	92.48	86.51	43.41
Chill+EBR1-1	41,024,790	40,319,076	5.04	0.04	94.22	88.8	43.63
Chill+EBR1-2	41,024,790	40,319,076	5.04	0.04	92.22	86.11	43.71
Chill+EBR2-1	38,701,387	37,690,638	4.71	0.04	95.02	90.2	42.95
Chill+EBR2-2	38,701,387	37,690,638	4.71	0.04	92.44	86.35	42.97

a *The numbers 1 and 2 at the end of the sample name represent left and right ends (pair-end sequencing), respectively. Chill-samples treated with water; Chill+EBR-samples treated with 0.1 μM EBR*.

b *Percentage of bases with a Phred value of at least 20*.

c *Percentage of bases with a Phred value of at least 30*.

d *Proportion of guanidine and cytosine nucleotides among total nucleotides*.

Each library that produced the clean reads was aligned to the recently released *C. annuum* reference genome, release_2.0 (Qin et al., 2014). The proportion of clean reads in the two pepper transcriptome libraries that mapped to *C. annuum* reference genome ranged from 85.34 to 87.60% (Table 2). A total of 39,829 genes were confirmed from the mapped libraries, including the locations of exons and introns (Table S2). All of the RNA-sequence data in this article have been deposited in the NCBI-SRA database and are accessible in SRX1959970.

**Table 2:** **Number of reads sequenced and mapped to the pepper genome**.

**Sample name**	**Chill1**	**Chill2**	**Chill+EBR1**	**Chill+EBR2**
Total reads	64,217,082	61,938,306	80,638,152	75,381,276
Total mapped	56,251,137 (87.6%)	53,309,676 (86.07%)	68,819,267 (85.34%)	65,985,060 (87.54%)
Multiple mapped	2,129,037 (3.32%)	2,064,848 (3.33%)	2,861,761 (3.55%)	28,11,891 (3.73%)
Uniquely mapped	54,122,100 (84.28%)	51,244,828 (82.74%)	65,957,506 (81.79%)	63173169 (83.8%)
Read-1	27,305,549 (42.52%)	25,918,892 (41.85%)	33,351,826 (41.36%)	32,030,587 (42.49%)
Read-2	26,816,551 (41.76%)	25,325,936 (40.89%)	32605680 (40.43%)	31,142,582 (41.31%)
Reads map to “+”	27,008,832 (42.06%)	25,526,323 (41.21%)	32,830,110 (40.71%)	31,512,085 (41.8%)
Reads map to “−”	27,113,268 (42.22%)	25,718,505 (41.52%)	33127396 (41.08%)	31,661,084 (42%)
Non-splice reads	36,188,485 (56.35%)	33853641 (54.66%)	43,682,428 (54.17%)	41,602,031 (55.19%)
Splice reads	17,933,615 (27.93%)	17,391,187 (28.08%)	22,275,078 (27.62%)	21,571,138 (28.62%)

### Transcriptome profiles of the leaves from the two groups of pepper seedlings

The 39,829 genes from the mapped libraries were normalized (Table S3) using the reads per kilo bases per million reads (RPKMs) method (Mortazavi et al., 2008). With RPKMs in 0 to 1, the genes were regarded as having a low expression level; genes with RPKMs of 3–15 were regarded as having a medium expression level; and genes with RPKM beyond 60 were regarded as having a very high expression level (Table 3).

**Table 3:** **Statistics of genes in different expression-level interval**.

**RPKM^a^ interval**	**Chill**	**Chill+EBR**
0 ~ 1	17,437 (43.78%)^b^	17,267 (43.35%)
1 ~ 3	3859 (9.69%)	3925 (9.85%)
3 ~ 15	8283 (20.80%)	8415 (21.13%)
15 ~ 60	6932 (17.41%)	6954 (17.46%)
>60	3319 (8.33%)	3269 (8.21%)

a *Reads per kilo bases per million reads*.

b *Ratios of gene number to total gene number are presented in parentheses*.

### Differentially expressed genes in the pepper leaves

**Figure 1:**
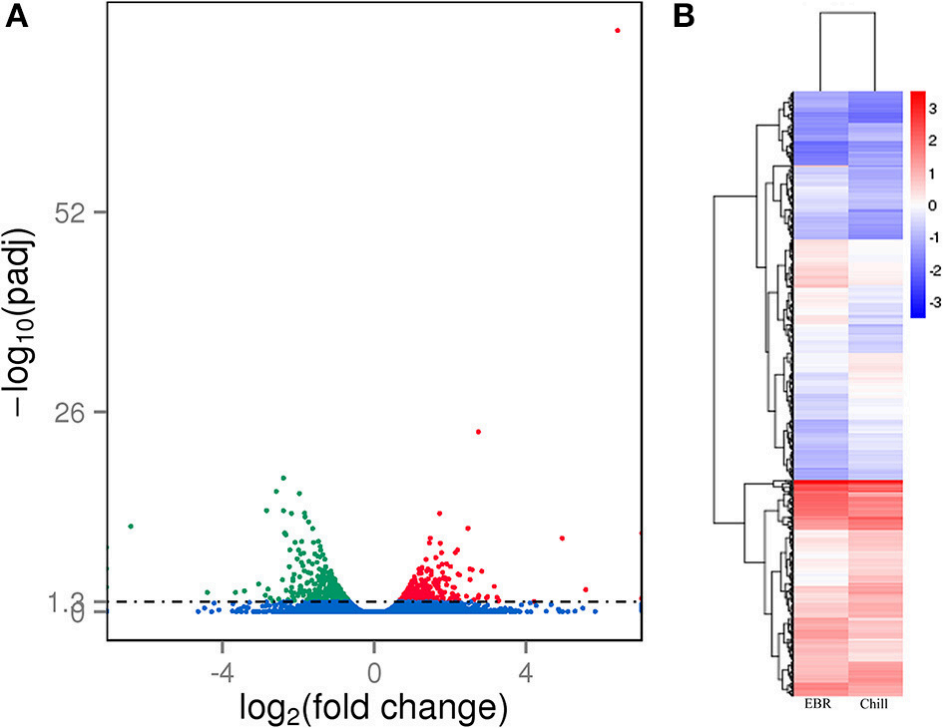
**Transcriptome analysis of differentially expressed genes in Chill and Chill+EBR treatment of pepper leaves. (A)** Volcano plot showed the DEGs between two different libraries. The *q* < 0.05 was used as thresholds to determine the significance of DEGs. Red dots represent up-regulated genes, green dots show down-regulated genes, and blue dots indicate transcripts that did not change significantly in the Chill+EBR library compared Chill. **(B)** Hierarchical clustering of all the DEGs based on log_10_ RPKM values. The color (from blue to red) represents gene expression intensity from low to high. Chill and Chill+EBR represent two treatments under alone chilling stress and chilling stress with 0.1 μM EBR.

We identified 656 DEGs between Chill and Chill+EBR treatments (Table S4; Figure 1A). We used hierarchical clustering of all the DEGs to observe the gene expression patterns, and it was evaluated by log_10_ RPKMs for the two groups (Figure 1B). Compared to Chill treatment, genes in Chill+EBR treatment contained 335 up-regulated genes and 321 down-regulated genes. These results suggest that EBR had a markedly effect on the transcription of a subset of genes response for chilling stress.

### Real-time qPCR analysis

**Figure 2:**
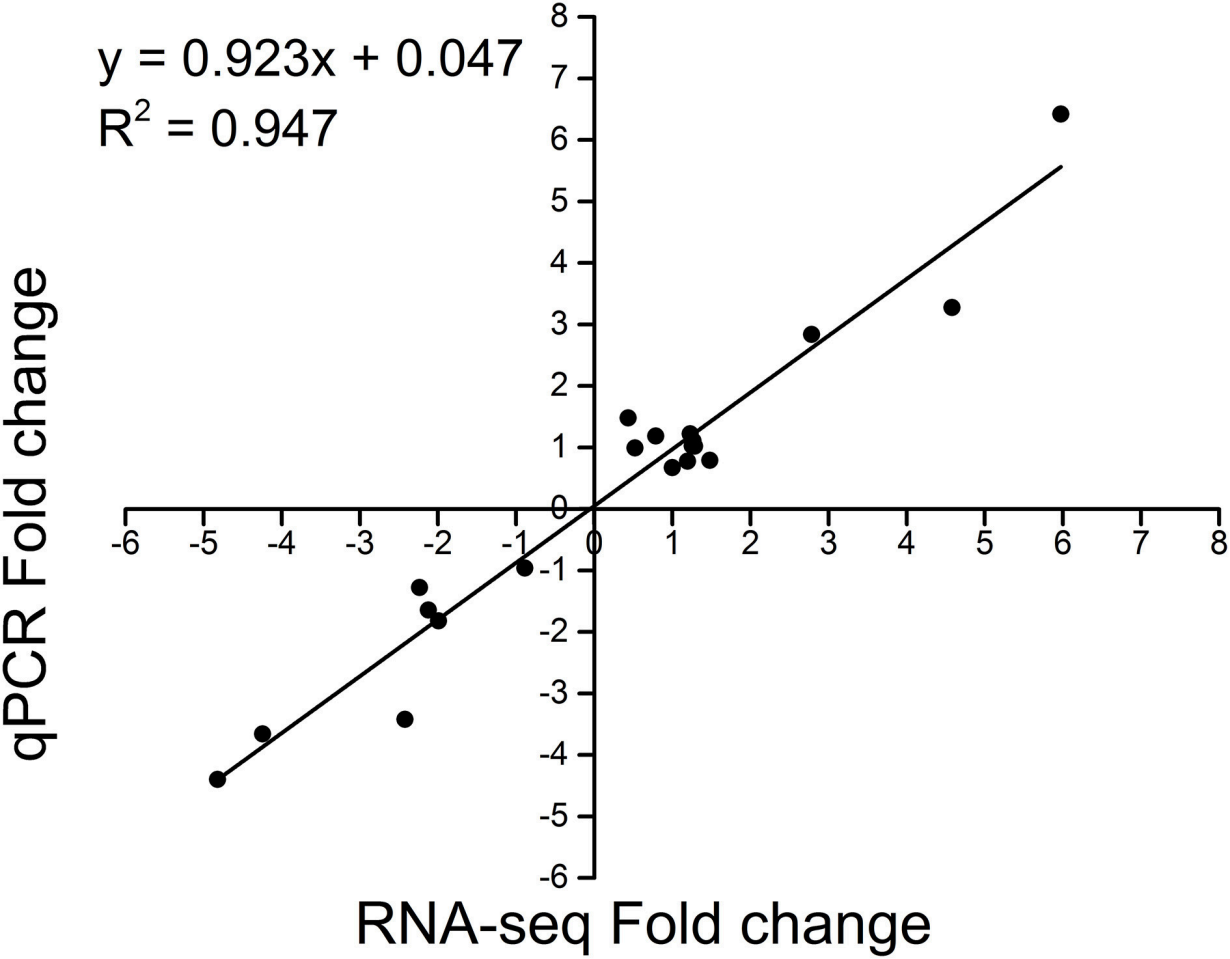
**Correlation of RNA-seq (y axis) and qRT-PCR data (x axis), and the assay is carried out for 20 randomly selected DEGs**. GO and KEGG enrichment analyses.

To validate the DEG data from RNA-sequencing, we randomly selected 20 DEG for qRT-PCR assay in EBR-mediated chilling stress. The qRT-PCR results showed a strong positive correlation with the RNA-seq (*R*^2^ = 0.947), indicating that the RNA-seq data were validated (Figure 2).

**Figure 3:**
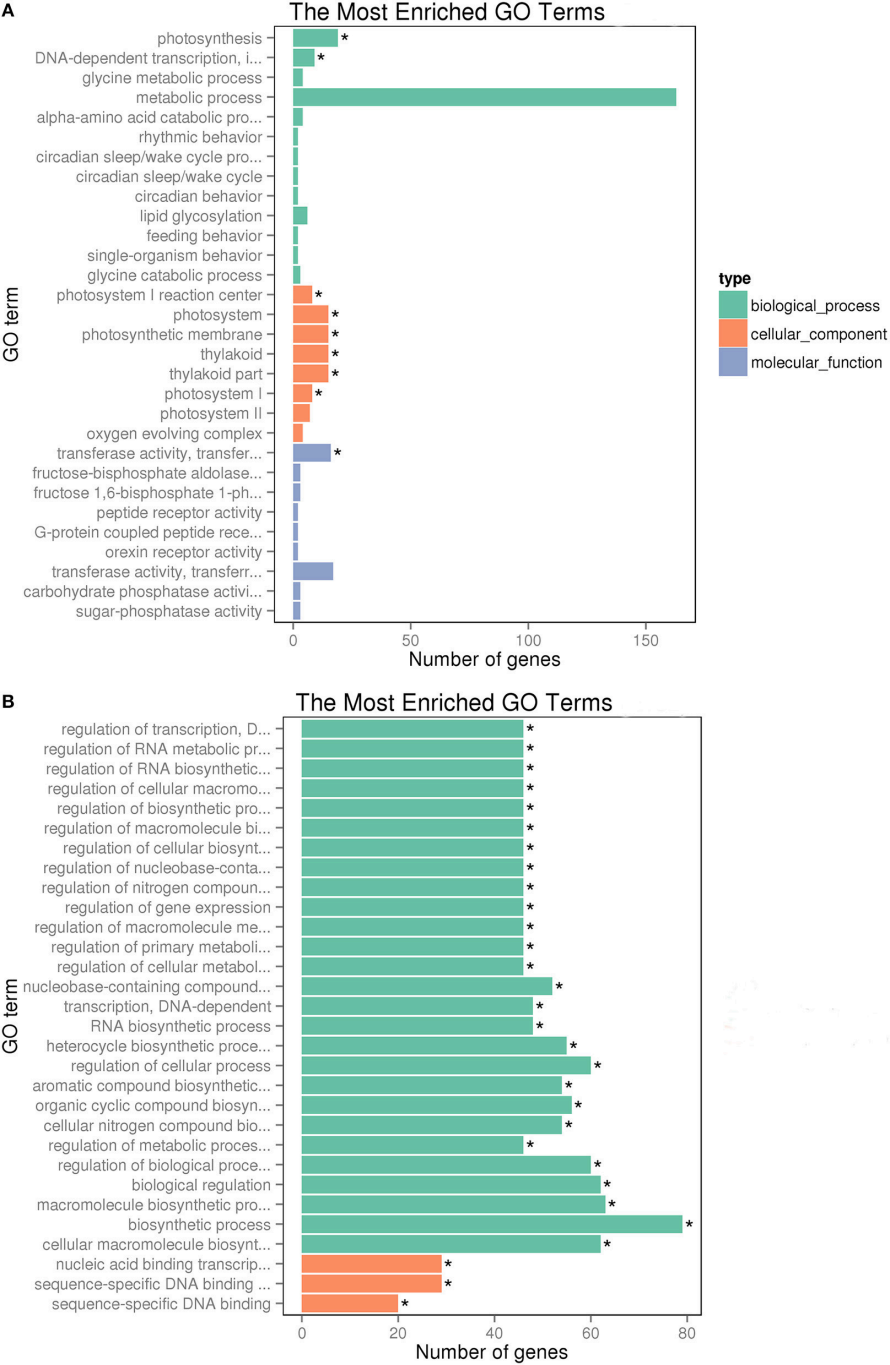
**GO enrichment analysis of DEGs between Chill and Chill+EBR treatment. (A)** up-regulation; **(B)** down-regulation. The 30 most enriched GO terms are shown. Asterisks indicate significantly enriched GO terms (*q* < 0.05).

We evaluated the gene functions of DEGs using GO enrichment analysis, and it revealed the biological process, molecular function and cellular component categories for the 656 DEGs of the two groups tested (Table S5). A total of 335 up-regulated DEGs were enriched significantly in 10 functional terms. Among these, six terms were significantly enriched in cellular component, there was one term significantly enriched in molecular function, and two terms were significantly enriched in biological process. Within in the cellular component domain, the terms that were significantly enriched Photosystem I reaction center (GO: 0009538; 8 genes), photosystem (GO: 0009521; 15 genes), photosynthetic membrane (GO: 0034357; 15 genes), thylakoid (GO: 0009579;15 genes), thylakoid part (GO: 0044436; 15 genes), and photosystem I (GO: 0009522; 8 genes). Within the molecular function domain, the term that was significantly enriched intransferring hexosyl groups (GO: 0016758) with 16 genes. Within the biological process domain, the terms that were significantly enriched included Photosynthesis (GO: 0015979) and DNA-dependent transcription (GO: 0006352) with 19 and 9 genes, respectively. For down-regulated DEGs, three terms were markedly enriched in molecular function, and 41 terms were markedly enriched in biological process (Figure 3).

**Figure 4:**
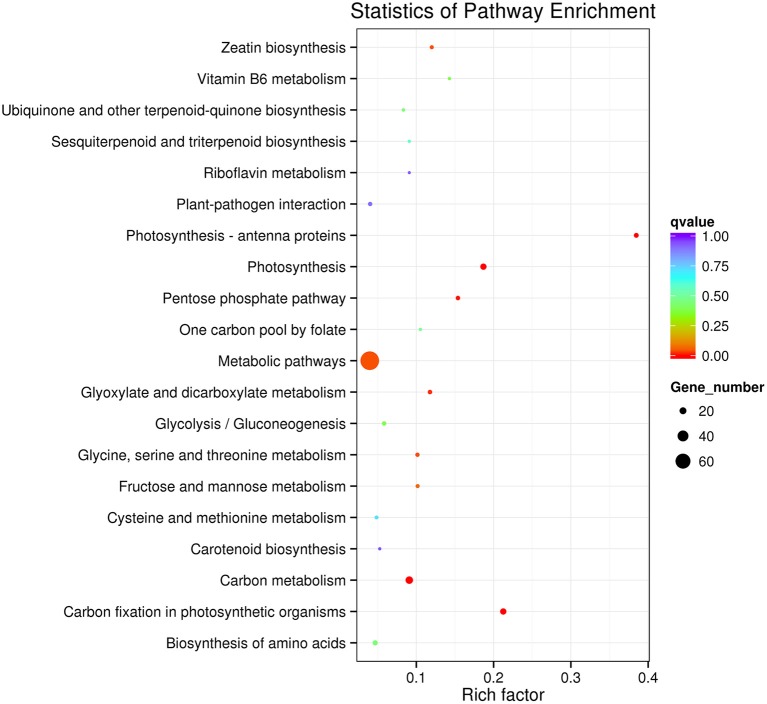
**KEGG pathway enrichment analysis of DEGs between Chill and Chill+EBR treatment**. The left Y-axis shows the KEGG pathway. The X-axis shows the Rich factor. A high *q*-value is represented by blue and a low *q*-value is represented by red (*q* < 0.05).

Among DGEs of the two groups tested the following 9 pathways, with a KEGG pathway annotation, were affected: carbon fixation in photosynthetic organisms, photosynthesis, photosynthesis-antenna proteins, carbon metabolism, pentose phosphate pathway, glyoxylate and dicarboxylate metabolism, metabolic pathways, zeatin biosynthesis, glycine, serine, and threonine metabolism pathway (*q* < 0.05; Table S6; Figure 4).

### Effect of EBR on chlorophyll content and net photosynthetic rate

**Figure 5:**
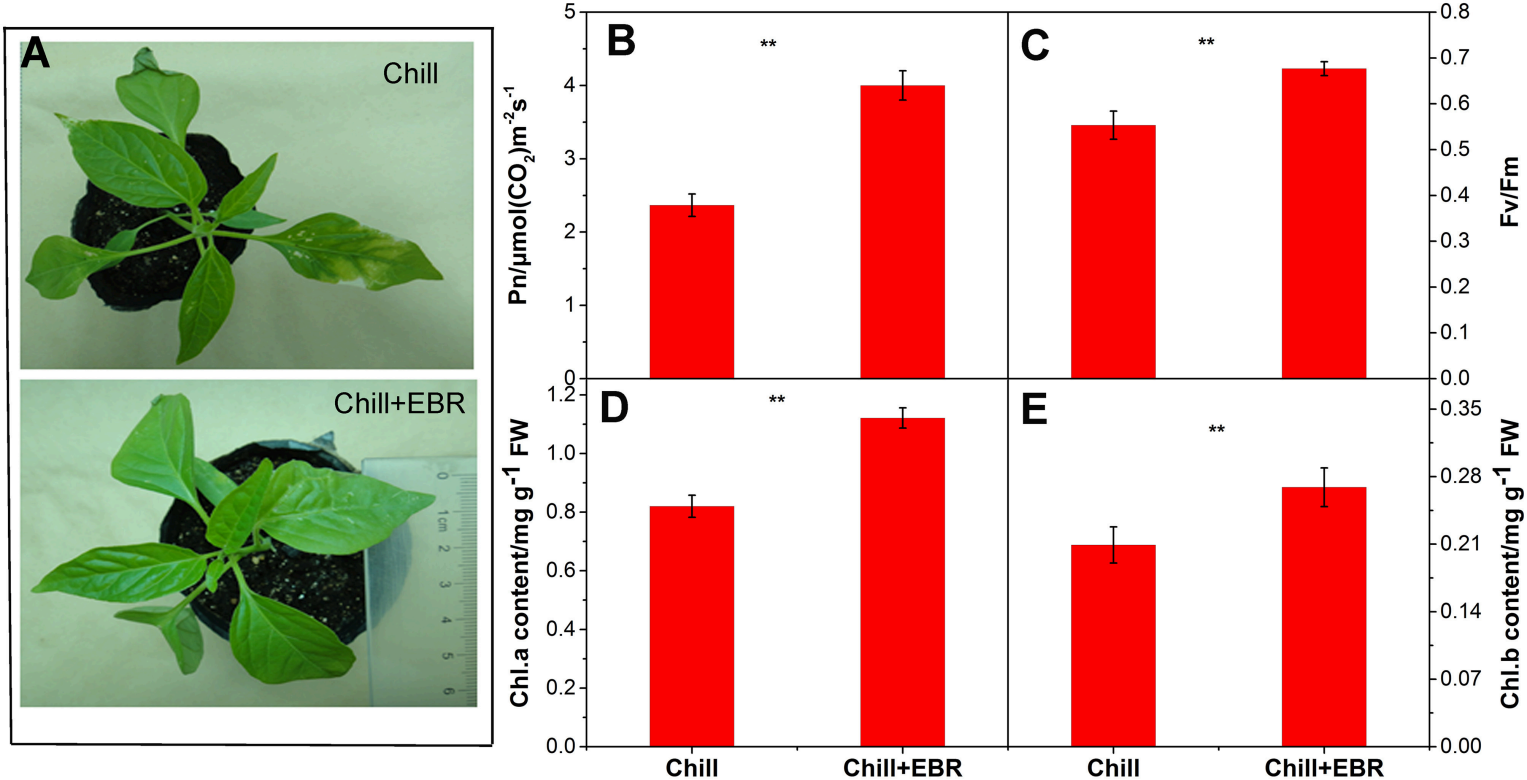
**Phenotypic changes (A), net photosynthetic rate (Pn) (B), Fv/Fm (C), chlorophyll a and chlorophyll b (D,E) in chill-stressed pepper seedlings with or without exogenous application of EBR**. Asterisks above the histograms indicated significant differences between Chill and Chill +EBT by Student's *t*-test (^**^P < 0.01; ^*^P < 0.05).

The exposure to chilling stress treatment influenced the morphological traits in pepper seedlings, and EBR application significantly alleviated the inhibited growth (Figure 5A). The net photosynthetic rate (Pn) was increased significantly 7 days after EBR-treatment compared to without EBR; this was accompanied by a remarkable increase in chlorophyll content (Figures 5D,E). EBR (Chill+EBR) attenuated the inhibitory effect of chilling on photosynthesis. Low temperature treatments resulted in the reduction of Fv/Fm in pepper seedlings, while the application of EBR significantly increased Fv/Fm (Figure 5C).

### Transcriptome profiles of photosynthesis-related genes

The genes involved in the photosynthesis in the EBR-treated chilling responses were all up-regulated, and among them 8 genes encoded photosystem I reaction center subunit. It is known that the chlorophyll a/b-binding protein is an important component of light-harvesting complex II (Jansson, 1999, 1994). We found that EBR induced the expression of 10 genes encoding chlorophyll a/b-binding protein. ATP-dependent zinc metalloproteases, known to take part in chloroplast protein modification and the metabolism of extracellular matrix, were also up-regulated by EBR. Additionally, the genes involved in ATP synthase chain, oxygen-evolving enhancer protein, ABC transporter I family member, thylakoid lumenal protein, and PsbP domain-containing protein were all up-regulated, suggesting that BRs might play a significant and positive role in the photosynthesis processes under chilling stress (Table 4).

**Table 4:** **Photosynthesis related genes expression in pepper leaves as influenced by chilling alone or in combination EBR treatment**.

**Gene ID**	**Log_2_FC**	***q*-value**	**Symbol**	**Description**
Capana06g001915	0.786	6.73E-03	*psaD*	Photosystem I reaction center subunit II
Capana02g001321	1.198	2.76E-02	*PSAF*	Photosystem I reaction center subunit III
Capana06g000155	1.229	3.90E-02	*PSAEA*	Photosystem I reaction center subunit IV A
Capana04g000192	0.798	6.55E-03	*PSAEA*	Photosystem I reaction center subunit IV A
Capana06g001274	1.170	1.58E-02	*PSAH*	Photosystem I reaction center subunit VI
Capana03g000290	0.700	2.66E-02	*PSAH*	Photosystem I reaction center subunit VI
Capana06g000231	0.733	1.60E-02	*PSAL*	Photosystem I reaction center subunit XI
Capana06g000232	1.184	2.77E-06	*PSAL*	Photosystem I reaction center subunit XI
Capana10g002492	1.216	2.83E-03	*PSBY*	Photosystem II core complex proteins psbY
Capana09g002353	1.130	2.36E-02	*psbW*	Photosystem II reaction center W protein
Capana09g000146	1.218	4.15E-02	*CB12*	Chlorophyll a/b binding protein
Capana07g001245	1.480	2.70E-10	*CAB4*	Chlorophyll a/b binding protein 4
Capana08g000250	1.106	1.82E-03	*CAB7*	Chlorophyll a/b binding protein 7
Capana08g001648	1.626	9.29E-08	*CAB8*	Chlorophyll a/b binding protein 8
Capana08g001647	1.834	4.99E-05	*CAB8*	Chlorophyll a/b binding protein 8
Capana00g002799	3.279	3.52E-02	*CAB21*	Chlorophyll a/b binding protein 21
Capana00g002801	2.960	1.58E-02	*CAB21*	Chlorophyll a/b binding protein 21
Capana09g000473	2.058	4.26E-03	*CAB37*	Chlorophyll a/b binding protein 37
Capana09g001520	0.956	2.74E-04	*LHCB4.2*	Chlorophyll a/b binding protein CP29.2
Capana01g002398	1.342	1.11E-03	*LHCB5*	Chlorophyll a/b binding protein CP26
Capana03g002052	0.915	1.06E-02	*FTSH11*	ATP-dependent zinc metalloprotease FTSH 11
Capana02g002193	0.690	2.66E-02	*ATPC*	ATP synthase gamma chain
Capana05g001562	0.768	2.19E-02	*ATPD*	ATP synthase delta chain
Capana00g001213	0.908	7.37E-04	*PSBP*	Oxygen-evolving enhancer protein 2
Capana02g002133	1.140	7.25E-04	*PSBQ2*	Oxygen-evolving enhancer protein 3-2
Capana06g001078	1.313	1.42E-02	*ABCI17*	ABC transporter I family member 17
Capana00g004571	1.295	1.83E-04	*At1g03600*	Thylakoid lumenal protein At1g03610
Capana04g000971	1.510	4.25E-03	*CLEB3J9*	Thylakoid lumenal 29 kDa protein
Capana06g001397	1.348	8.68E-05	*PPD6*	PsbP domain-containing protein 6

*Shown are the q-value (< 0.05) for genes expression (comparison between EBR+Chill and Chill)*.

### Effect of EBR on the endogenous levels of other hormones and their metabolites

**Figure 6:**
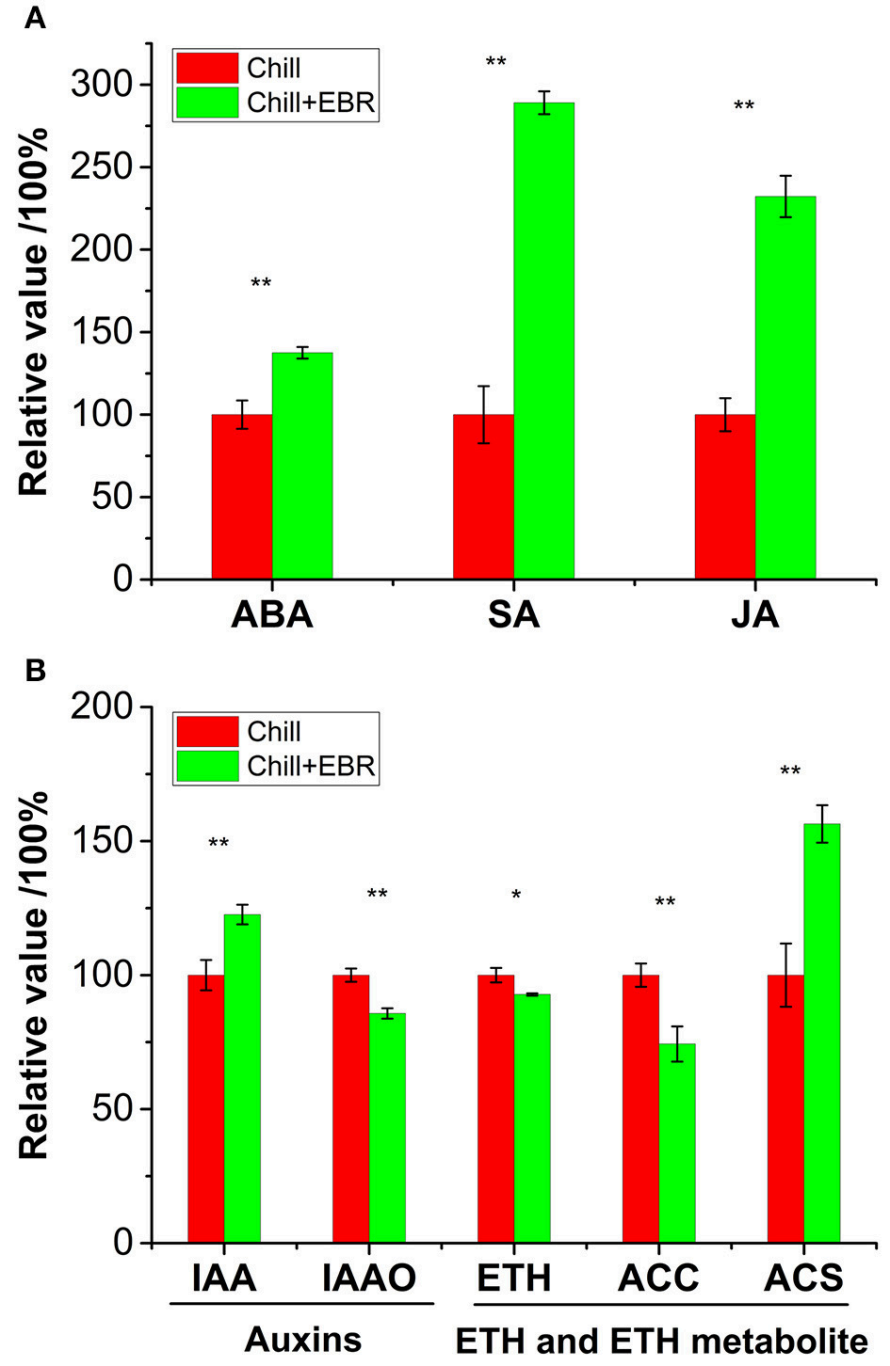
**Endogenous hormone profiles of pepper seedlings grown in the only chill stress (Chill) or chill stress with 0.1 μM EBR (Chill+EBR)**. **(A)** Contents of ABA, SA and JA in leaves of pepper seedlings. **(B)** Contents and metabolites of IAA and ETH in leaves of pepper seedlings. Error bars represent the standard error (SE) of the mean for three replicates. Asterisks above the histograms indicated significant differences between Chill and Chill +EBT by Student's *t*-test (^**^P < 0.01; ^*^P < 0.05).

To determine whether BR influenced the endogenous levels of other hormones, we assessed the concentrations of IAA, ABA, JA, and SA and ETH metabolites in the leaves of pepper seedlings grown in the absence or presence of 0.1 μM EBR under chilling stress. The concentrations of ABA, SA and JA were significantly increased by 37.5, 189.1, and 132.3%, respectively, in the EBR-treated seedlings compared to the untreated seedlings (Figure 6A). There was an increase in the level of IAA, but a significant decrease in the activity of acetic acid oxidase (IAAO) in the EBR-treated tissue (Figure 6B). There was a significant increase in the activity of ACC synthase (ACS) in the EBR-treated seedlings compared to chilling stress without EBR (Figure 6B).

### Transcriptome profiles of hormone metabolism and signaling-related genes

EBR treatment under chilling stress induced changes in the expression of some genes involved in auxin signaling pathways, including auxin-induced protein, auxin-responsive protein, and auxin-binding protein (Table 5). Genes involved in cytokinin dehydrogenase, which plays a significant role in maintaining the well-organized cytokinin functions (Werner et al., 2003), were down-regulated by EBR in pepper. Protein phosphatase type 2C24 (*P2C24*) was identified as the second component of the ABA signaling pathway, which was also down-regulated by EBR. JA biosynthesis gene, linoleate 13S-lipoxygenase 2-1, and SA-related isochorismate synthase gene were all up-regulated, while four gibberellin-responsive genes were down-regulated. EBR treatment under chilling stress down-regulated all of the genes in the ETH signaling pathways. These genes included the ETH biosynthesis gene ACC synthase (*ACS1*), the induction of the ETH receptor (*ETR*), 8 ETH responsive transcription factors and one ETH insensitive 3-like 1 protein.

**Table 5:** **Hormone metabolism and signaling-related gene expression in pepper leaves as influenced by chilling alone or in combination EBR treatment**.

**Gene ID**	**Log_2_FC**	***q*-value**	**Symbol**	**Description**
**AUXIN**				
Capana09g001771	−1.705	9.66E-03	Unknown	Auxin-induced protein 6B
Capana03g004288	1.079	6.65E-03	Unknown	Auxin-induced protein PCNT115
Capana03g000310	−0.888	1.67E-03	*IAA17*	Auxin-responsive protein IAA17
Capana07g000961	1.786	2.76E-02	*ABP19A*	Auxin-binding protein ABP19a
**CYTOKININ**				
Capana00g003808	−1.856	1.99E-02	*CKX6*	Cytokinin dehydrogenase 6
**ABSCISIC ACID**				
Capana03g002801	−1.084	2.66E-02	*P2C24*	Probable protein phosphatase 2C 24
**JASMONIC ACID**				
Capana01g001577	6.419	2.41E-76	*LOX2.1*	Linoleate 13S-lipoxygenase 2-1
**SALICYLIC ACID**				
Capana06g001004	1.021	4.65E-02	*ICS*	Isochorismate synthase
**GIBBERELLIN**				
Capana03g001010	−2.357	5.88E-11	*GASA14*	Gibberellin-regulated protein 14
Capana01g002809	−1.463	0.011357	*GA2OX2*	Gibberellin 2-beta-dioxygenase 2
Capana03g001010	−2.357	5.88E-11	*GASA14*	Gibberellin-regulated protein 14
Capana05g000709	−2.415	7.10E-05	*GA2OX7*	Gibberellin 2-beta-dioxygenase 8
**ETHYLENE**				
Capana01g000175	−1.589	1.73E-02	*ACS1*	1-aminocyclopropane-1-carboxylate synthase
Capana03g004532	−1.636	4.05E-02	*ETR2*	Ethylene receptor 2
Capana06g000678	−0.805	7.97E-03	*EIL1*	Ethylene insensitive 3-like 1 protein
Capana00g003399	−0.945	2.40E-03	*ERF061*	Ethylene-responsive transcription factor ERF061
Capana01g000662	−1.770	2.69E-02	*ERF5*	Ethylene-responsive transcription factor 5
Capana03g000085	−0.718	2.11E-02	*RAP2-12*	Ethylene-responsive transcription factor RAP2-12
Capana04g001107	−0.941	8.75E-04	*RAP2-4*	Ethylene-responsive transcription factor RAP2-4
Capana05g001701	−1.143	2.51E-05	*ERF1B*	Ethylene-responsive transcription factor 1B
Capana05g001951	−1.478	6.45E-08	*ERF106*	Ethylene-responsive transcription factor ERF106
Capana07g001714	−1.603	4.93E-03	*ERF4*	Ethylene-responsive transcription factor 4
Capana10g000499	−1.353	3.63E-08	*ERF4*	Ethylene-responsive transcription factor 4
**OTHER SIGNALING-RELATED GENE**				
Capana02g002718	−3.045	2.50E-04	*LEA29*	Late embryogenesis abundant protein D-29
Capana00g002896	−0.777	1.00E-02	*POX2*	Proline dehydrogenase 2, mitochondrial
Capana01g002720	−0.997	3.08E-04	*PERK1*	Proline-rich receptor-like protein kinase PERK1
Capana04g000460	1.357	1.36E-04	*PERK10*	Proline-rich receptor-like protein kinase PERK10
Capana01g004123	−2.109	3.57E-03	Unknown	14 kDa proline-rich protein DC2.15
Capana03g000595	−1.306	7.23E-05	*RBOHC*	Respiratory burst oxidase homolog protein C
Capana12g000877	−1.355	3.59E-02	*CRK25*	Cysteine-rich receptor-like protein kinase 25
Capana12g000878	−0.936	4.87E-02	*CRK10*	Cysteine-rich receptor-like protein kinase 10
Capana00g004816	2.524	2.82E-06	*CRK25*	Cysteine-rich receptor-like protein kinase 25
Capana06g002351	0.851	4.51E-03	*ATL72*	RING-H2 finger protein ATL72
Capana00g003879	−1.036	4.84E-02	*ATL78*	RING-H2 finger protein ATL78
Capana01g002369	−1.149	5.76E-05	*ATL2*	RING-H2 finger protein ATL2
Capana10g001323	−1.6687	9.52E-06	*HSP18.1*	18.1 kDa class I heat shock protein (Fragment)
Capana05g002063	−0.9636	0.013016	*DREB2C*	Dehydration-responsive element-binding protein
Capana01g002345	−0.89067	0.02905	*DI19-3*	Protein Dehydration-induced 19 homolog 3
Capana00g003859	−1.3668	0.02239	*At5g39030*	Probable receptor-like protein kinase At5g39030
Capana08g002140	−0.8267	0.01623	*IKU2*	Receptor-like protein kinase HAIKU2
Capana12g002105	0.941	5.94E-06	*TMK1*	Probable receptor protein kinase TMK1

*Shown are the q-value (< 0.05) for genes expression (comparison between EBR+Chill and Chill)*.

Late embryogenesis abundant protein D-29, which is involved in the tolerance to water stress resulting from desiccation or chilling injury was down-regulated by EBR. The genes involved in proline responses including proline dehydrogenase and proline-rich receptor-like protein kinase PERK1 (*PERK1*) were down-regulated, while *PERK10* was up-regulated. *RBOHC* which is associated with ROS production in plants, was down-regulated by EBR. There are three genes related to cysteine-rich receptor-like protein kinase, among them two genes were down-regulated, and the other gene was up-regulated. The RING-H2 finger protein ATL72 was up-regulated, while the RING-H2 finger protein ATL78 and RING-H2 finger protein 2 were down-regulated. The genes may be involved in the early steps of the plant defense signaling pathway. Dehydration-responsive element-binding protein (*DREB2C*) and protein dehydration-induced 19 homolog (*3DI19-3*) which mediate cold-inducible transcription, were down-regulated by BRs (Table 5).

### Transcriptome profiles of calcium signaling and redox-related genes

We found that EBR treatment under chilling stress up-regulated calcium-dependent protein kinase (*CAS*, Capana00g001365), while CBL-interacting serine/threonine-protein kinase, calcium uniporter protein, probable calcium-binding protein CML15, calcium-binding protein CML38, and calmodulin-related protein were down-regulated (Table 6).

**Table 6:** **Calcium signaling and redox-related genes expression in pepper leaves as influenced by chilling alone or in combination EBR treatment**.

**Gene ID**	**Log_2_FC**	***q*-value**	**Symbol**	**Description**
**CALCIUM**				
Capana00g001365	0.727	1.88E-02	*CAS*	Calcium sensing receptor
Capana06g000284	−1.279	1.77E-06	*CIPK11*	CBL-interacting serine/threonine-protein kinase 11
Capana02g000787	−1.128	1.06E-05	*MCU*	Calcium uniporter protein
Capana03g000955	−1.945	4.74E-04	*CML15*	Probable calcium-binding protein CML15
Capana11g000436	−1.920	3.75E-07	*CML38*	Calcium-binding protein CML38
Capana10g002124	−1.084	8.67E-05	*CAM53*	Calmodulin-related protein
**REDOX**				
Capana09g001740	1.765	8.55E-03	*GSTX1*	Probable glutathione S-transferase
Capana02g002747	2.8381	5.52E-06	*PER72*	Peroxidase 72
Capana02g002452	1.149	4.89E-02	*CAT2*	Catalase isozyme 2
Capana04g000138	0.884	1.36E-03	*CDSP32*	Thioredoxin-like protein CDSP32
Capana01g004227	2.526	6.43 E-05	*FAO4A*	Long-chain-alcohol oxidase FAO4A
Capana00g002845	3.276	4.01E-02	Unknown	NAD(P)H:quinone oxidoreductase
Capana03g001927	1.616	1.49E-04	*At1g06690*	Uncharacterized oxidoreductase, chloroplastic

*Shown are the q-value (< 0.05) for genes expression (comparison between EBR+Chill and Chill)*.

All of the genes involved in redox homeostasis were up-regulated by EBR (Table 6). *GSTX1, PER72*, and *CAT2* inactivate endogenous epoxides and hydroperoxides, and are associated with secondary metabolites during oxidative stress. *CDSP32*, which encodes thioredoxin as a physiological electron donor to the BAS1 peroxiredoxin, participated in the defense against lipid peroxidation in photosynthetic membranes (Broin and Rey, 2003). *FAO4A* was involved in the omega-oxidation pathway of lipid degradation. *At1g06690* and NAD(P)H quinone oxidoreductase played a positive role in the antioxidant defense by generating vitamin E and ubiquinone.

### Transcriptome profiles of transcription factors and post transcription

We analyzed the function of transcription factors (TFs) in pepper based on their annotations in NCBI database. The majority of WRKY family TFs were down-regulated, such as *WRKY11* [a negative regulator of resistance to *Pst* (Journot-Catalino et al., 2006)], *WRKY41, WRKY6, WRKY40* [a transcriptional repressor in plant cells response to abscisic acid and abiotic stress (Chen et al., 2010)], *WRKY33, WRKY28*, and *WRKY20*. Only *WRKY51* (Capana12g001826), which positively mediated the signaling transduction of SA and JA (Gao et al., 2011), was up-regulated by EBR under chilling stress. The MYB family, which is involved in a diversity of gene regulation (Jackson et al., 1991), and NAC family members (*NAC100, NAC002*, and *NAC072*) were all down-regulated by EBR under chilling stress. bHLH family members were up-regulated except *bHLH130*. bHLH transcription factors positively regulated ABA-responsive kinase substrate (AKS) and facilitated stomatal opening by triggering the phosphorylation of AKS family transcription factors (Takahashi et al., 2013). Heat-stress transcription factor A-2/B-1, the key regulator alleviating oxidative damage caused by heat stress negatively regulated by cold stress in Arabidopsis (Zhang et al., 2009), was down-regulated by EBR in pepper. *COL2*, a circadian clock that controls many rhythmic processes (Kim et al., 2013), was up-regulated. *MGP*, which regulated tissue boundaries and asymmetric cell division and controlling SHORT-ROOT activity in a transcriptional and protein interaction network (Welch et al., 2007), was down-regulated by EBR. Additionally, *ATHB-7* mediates a drought response via transcriptional regulation in an ABA-dependent manner. *ATHB-52* and *ATHB-21*, which responded to auxin, ETH, and ABA or water deficits (Henriksson et al., 2005), were down-regulated by EBR. *TCP19*, involved in the orchestrated regulation of *ICS1* expression (Wang X. et al., 2015), was down-regulated by EBR. *GATA22*, which participates in the GA-mediated signaling pathway (Richter et al., 2010) and chlorophyll biosynthetic process (Hudson et al., 2011), was up-regulated by EBR in pepper under chilling stress (Table 7).

**Table 7:** **Transcription factors and post-transcription related genes expression in pepper leaves as influenced by chilling alone or in combination EBR treatment**.

**Gene ID**	**Log_2_FC**	***q*-value**	**Symbol**	**Description**
**TRANSCRIPTION FACTORS**				
Capana00g003083	−1.152	4.21E-04	*WRKY11*	Probable WRKY transcription factor 11
Capana01g004472	−1.627	5.85E-07	*WRKY41*	Probable WRKY transcription factor 41
Capana02g002230	−1.123	9.79E-05	*WRKY6*	WRKY transcription factor 6
Capana03g000473	−1.909	7.33E-10	*WRKY40*	Probable WRKY transcription factor 40
Capana06g001110	−1.726	2.04E-12	*WRKY40*	Probable WRKY transcription factor 40
Capana06g001506	−1.139	5.55E-03	*WRKY33*	Probable WRKY transcription factor 33
Capana09g001251	−1.221	2.94E-05	*WRKY33*	Probable WRKY transcription factor 33
Capana07g001968	−1.071	8.10E-03	*WRKY28*	Probable WRKY transcription factor 28
Capana07g002350	−1.064	8.71E-05	*WRKY20*	Probable WRKY transcription factor 20
Capana12g001826	1.562	3.98E-02	*WRKY51*	Probable WRKY transcription factor 51
Capana02g002068	−1.643	8.43E-05	*MYB39*	Transcription factor MYB 39
Capana02g003369	−1.485	2.37E-05	*MYB306*	Myb-related protein 306
Capana03g000766	−1.099	4.99E-05	*MYB306*	Myb-related protein 306
Capana01g000650	−1.645	8.28E-05	*NAC100*	NAC domain-containing protein 100
Capana03g000802	−0.837	2.61E-02	*NAC100*	NAC domain-containing protein 100
Capana05g000569	−1.148	1.47E-05	*NAC002*	NAC domain-containing protein 2
Capana07g002219	−0.891	1.24E-03	*NAC072*	NAC domain-containing protein 72
Capana09g000936	−0.804	4.78E-03	*NAC072*	NAC domain-containing protein 72
Capana01g002561	−0.695	4.11E-02	*bHLH130*	Transcription factor bHLH130
Capana02g000436	1.652	1.35E-02	*bHLH64*	Transcription factor bHLH64
Capana03g002644	1.181	7.40E-03	*bHLH51*	Transcription factor bHLH51
Capana10g000647	1.036	1.36E-03	*bHLH14*	Transcription factor bHLH14
Capana02g001490	−1.427	1.75E-02	*HSFB1*	Heat stress transcription factor B-1
Capana07g000898	−1.210	6.80E-05	*HSFA2*	Heat stress transcription factor A-2
Capana02g003199	1.028	3.06E-02	*COL2*	Zinc finger protein CONSTANS-LIKE 2
Capana04g000388	−0.939	1.23E-02	*MGP*	Zinc finger protein MAGPIE
Capana01g000046	−1.858	2.34E-08	*ATHB-7*	Homeobox-leucine zipper protein ATHB-7
Capana03g001926	−0.701	3.71E-02	*ATHB-7*	Homeobox-leucine zipper protein ATHB-7
Capana03g002675	−0.865	2.69E-02	*ATHB-52*	Homeobox-leucine zipper protein ATHB-52
Capana04g002305	−1.170	1.44E-05	*ATHB-21*	Homeobox-leucine zipper protein ATHB-21
Capana01g000353	−0.935	2.89E-02	*TCP19*	Transcription factor TCP19
Capana09g000221	2.084	6.49E-04	*GATA22*	Putative GATA transcription factor 22
**POST- TRANSCRIPTION**				
Capana05g002235	1.140	3.19E-03	*CSLE6*	Cellulose synthase-like protein E6
Capana07g001101	−0.727	2.42E-02	*CSLG1*	Cellulose synthase-like protein G1
Capana07g001384	0.906	1.45E-02	*CSLH1*	Cellulose synthase-like protein H1
Capana02g001405	−1.297	1.03E-04	*UGT91C1*	UDP-glycosyltransferase 91C1
Capana06g000316	1.161	3.07E-02	*UGT87A2*	UDP-glycosyltransferase 87A2
Capana10g001522	1.364	1.56E-07	*UGT73C3*	UDP-glycosyltransferase 73C3
Capana10g001627	1.965	5.76E-05	*UGT76E1*	UDP-glycosyltransferase 76E1
Capana11g001875	1.222	2.33E-02	*GT6*	UDP-glucose flavonoid 3-O-glucosyltransferase 6
Capana12g000737	1.058	3.71E-02	*GT7*	UDP-glucose flavonoid 3-O-glucosyltransferase 7
Capana12g002768	0.968	1.53E-03	*GT3*	UDP-glucose flavonoid 3-O-glucosyltransferase 3
Capana06g002960	1.187	1.08E-05	*GT4*	Putative UDP-rhamnose:rhamnosyltransferase 1
Capana10g000708	−0.976	6.90E-04	*UPTG2*	Alpha-1,4-glucan-protein synthase [UDP-forming] 2

*Shown are the q-value (< 0.05) for genes expression (comparison between EBR+Chill and Chill)*.

EBR treatment induced *CSLE6* and *CSLH1* up-regulation, but down-regulated *CSLG1*, and it is known that they encode various non-cellulosic β-linked polysaccharides synthesis enzymes involved in the backbone of the cell wall (Doblin et al., 2009). Four genes encoding glycosyltransferase, which catalyzed the receptor substrates of cytokinin, auxin and ABA, were up-regulated. Similarly, three genes encoding UDP-glucose flavonoid 3-O-glucosyltransferase, *GT6, GT7*, and *GT3*, were up-regulated by EBR (Table 7), and it is known that they are also involved in the detoxification of xenobiotics.

## Discussion

BRs, a group of naturally occurring plant steroids, are involved in many important cellular and physiological processes of plants in response to environmental stresses (Xia et al., 2009). Additionally, BRs mediate the response and signal transduction pathways of multiple hormones such as salicylic acid (SA), ethylene (ETH), jasmonic acid (JA), or abscisic acid (ABA) to abiotic stresses (U. K. Divi et al., 2010). In a previous study, we demonstrated that EBR treatment increased the basal chill tolerance of pepper via physiological and biochemical methods (Li et al., 2015a). To identify BR-mediated changes in gene expression, in the present study, we revealed the potent role of BRs in the response to chilling stress at the transcriptome level, and the findings provide the groundwork for elucidating the mechanism of BRs-induced chilling tolerance in pepper plants. We identified 656 DEGs, including 335 up-regulated and 321 down-regulated genes in the EBR-treated tissues compared to the chilling stress without EBR.

Photosynthesis is among the primary processes in plants that are often affected by chilling stress (Allen and Ort, 2001). In the present study, we used GO enrichment analysis and found that the 29 genes involved in photosynthesis were up-regulated by EBR under chilling stress conditions. Indeed, EBR-treated seedlings under chilling stress significantly enhanced net photosynthetic rate (Pn) and chlorophyll content when compared to controls (Figure 5). This suggests that BRs induction of chlorophyll and the net photosynthetic rate is a major factor contributing to the distinct photosynthetic characteristics in the BRs transcriptome. We identified several up-regulated genes associated with chloroplast organization (Capana02g002385) and the photosynthetic apparatus including the chloroplast and thylakoid luminal. In addition, we found that BRs also significantly increased Fv/Fm under chilling stress. These results suggest that EBR treatment contributes to the pepper leaves increased ability to absorb and transfer light energy in chilling conditions. Indeed, the 5 up-regulated genes were associated with redox regulation of photosystem II (Capana06g001397), transport activity (Capana06g001078, Capana03g002052), and photosystem II reaction center (Capana09g002353, Capana10g002492). KEGG pathway analysis also showed that the photosynthesis (ko00195) and photosynthesis-antenna proteins (ko00196) were significantly enriched up-regulated pathway terms (Figure 4).

As small molecules, plant hormones mediate many cellular processes in plants, including plant morphogenesis, and responses to changing environmental conditions (Kang et al., 2005; Pantin et al., 2013; Colebrook et al., 2014). Signal transduction components perceive plant hormone signals and transmit to the nuclear to induce gene expression synergistically with other signals to induce gene expression synergistically with other signals to influence response to environmental stress via a series of physiological processes (Lu et al., 2014). In our study, some unigenes were markedly enriched in the hormone biosynthesis and signaling components processes. These hormones including auxin, JA, ETH, ABA, and SA, and their signaling components were involved in BR-induced plant defense. Our results showed that the auxin, JA, and SA pathways appeared to act synergistically with BRs in mediating the response to chilling stress (Table 5). It is known that BRs can induce the SA-perceptive pathway or ABA-dependent pathways for resistance to heat and salt stress in *A. thaliana* or *Chlorella vulgaris* (Bajguz, 2009; Divi et al., 2010).

We demonstrated the effects of BRs on chilling tolerance via the transcriptome profiles of hormone metabolism and signaling-related components in pepper. Accordingly, the transcription involved in hormone signaling components may function as important mediators of BR-induced resistance to chilling stress. We found that four DEGs were associated with auxin biosynthesis and signaling pathways, and the BRs mediated the down-regulation of auxin-induced protein B6 and *IAA17*. It is known that auxin-induced protein B6 induced the auxin-activated signaling pathway, whereas *IAA17* functioned as a repressor of early auxin response genes (Liscum and Reed, 2002). Furthermore, we found that EBR-treated seedlings had significantly enhanced IAA content, but decreased the activity of acetic acid oxidase (IAAO). It was speculated that BRs reduced sensitivity to IAA by down-regulating genes encoding IAA conjugates. We found the down-regulation of genes involved in cytokinin; EBR treatment did not affect the endogenous levels of cytokinins (data not shown), but it induced cytokinin signaling in pepper. We speculated that BRs have a negative feedback loop to ensure the proper regulation of cytokinin functions, supported by previous observations (Werner et al., 2003). In addition, we found that ABA levels were increased by 37.5% in EBR-treated seedlings (Figure 6). Intriguingly, the gene *P2C24*, encoding the second component of the ABA signaling pathway, was down regulated. Indeed, Yu et al. (2011) demonstrated some ABA response genes as the direct targets of BES1 in ChIP-chip analysis. Additionally, BRs can inhibit ABA effects. For example, BRs acted as an opposing factor, providing inhibitory effects of ABA on seed germination (Divi and Krishna, 2010). The molecular mechanism is that BRs inhibit BRASSINOSTEROID INSENSITIVE2 (BIN2), thus, repressing the BIN2-ABI5 (ABSCISIC ACID INSENSITIVE5) cascade and antagonizing ABA inhibitory effect on germination (Hu and Yu, 2014).

We found that JA and SA levels were significantly higher in the EBR-treated seedlings (Figure 6). Likewise, the JA biosynthesis gene, linoleate 13S-lipoxygenase 2-1, and SA-related gene, isochorismate synthase (*ICS*), were up-regulated. These results suggest that the possible cross-talk of BRs with JA and SA signaling pathways plays a positive role of mediating chilling stress responses in pepper. In the present study, we found that EBR treatment decreased ETH levels, reduced the content of ACC which is an ETH synthetic substance, and increased ACS activity. We also identified genes encoding ETH signaling components (*ACS1*) and encoding ETH-insensitiive 3-like protein and ethylene-responsive transcription factors (*ERF*), which were down-regulated by EBR. This finding indicates that BRs and ETH have an antagonistic relationship under chilling stress and that ETH contributes to the activation of the BR signaling pathway and increased chill tolerance.

As secondary signaling molecules, ROS and calcium are crucial for plant defense against abiotic stresses. Here, we found that EBR up-regulated genes associated with cellular redox homeostasis, including glutathione S-transferase (*GSTX1*), peroxidase, catalase isozyme, and ferredoxin related genes (Table 6). It was reported that RBOHs are associated with ROS production in plants (Marino et al., 2012). We found that BRs triggered *RBOHC* down-regulation in pepper, which suggested that BRs reduced apoplastic ROS accumulation generated by NADPH oxidase so that the plants increased stress tolerance. Calcium is a key element in many cellular processes in plants, and calcium signaling is crucial for plant defense against abiotic stresses (Yuan et al., 2007; Boudsocq and Sheen, 2013). In our study, EBR up-regulated *CAS*, which modulated cytoplasmic Ca^2+^ concentrations for the induction of a series of biochemical reactions to chilling stress (Table 6). In addition, EBR-treated down-regulated the genes that encoded CBL-interacting serine/threonine-protein kinase, calcium-binding protein CML, and calmodulin-related protein. It was most likely that BR induced calmodulin-related signaling transduction, and Ca^2+^ was pumped quickly out of the cells and then returned to the Ca^2+^ pools through intracellular Ca^2+^-ATPase.

Transcriptional facts (TFs) are very important for the combination of cis acting element in gene promoter elements and the mediation of the signaling pathway in response to stresses, such as WRKYs, MYBs, NACs, BHLHs, and ZFPs (Liu et al., 2008; Fujita et al., 2011; Shi and Chan, 2014; Shi et al., 2014). Several studies have shown that the expressions of C-repeat binding factors (CBFs) as master molecular switches are positively regulated by *MYB56, ZFP1/182*, and *CAMTA1/2/3* and are negatively regulated by *MYB15, MYBS3, WRKY34*, and *EIN3*. The TFs specifically bind to the DRE/CRT (dehydration-responsive element/C-repeat element) *cis*-acting regulatory element of the promoter region of the cold-responsive genes, such as *DHN* (dehydrin) and *RD* (responsive to dehydration). In the present study, we found that BRs down-regulated *WRKY11* and *WRKY40* which mediated the metabolic pathway of ABA, whereas *WRKY51*, which positively mediated JA- and SA-signaling was the up-regulated by EBR under chilling stress. Additionally, BRs induced up-regulation of TFs bHLH involved in AKS, which facilitated stomatal opening by triggering the phosphorylation of AKS family transcription factors. We also found that BRs positively mediated *TCP19* TF, which was associated with the orchestrated regulation of *ICS1* expression and the GA-mediated signaling pathway (*GATA22*). A study on blueberry demonstrated that the genes encoding zinc finger proteins are associated with cold acclimation (Die and Rowland, 2014). In pepper, we found that EBR up-regulated *COL2* and down-regulated *MGP* under chilling stress. These results indicate that BRs induced different expressions of zinc finger proteins in response to chilling stress. Although it was is unknown why some TFs are up-regulated while the others are down-regulated in DEGs, we assume that TFs coordinated the network regulation of transcriptional activities in multiple pathways to increase chilling tolerance in EBR-treated pepper.

Glycosylation in plants is crucial not only for the regulation of cellular metabolism including plant hormones, secondary metabolites, and xenobiotics (Li et al., 2001), but also for the activity of several signaling molecules and defense compounds (Vogt and Jones, 2000). Sun et al. (2013) reported that glycosyltransferase could protect tobacco against salt stress, and the corresponding glycosyltransferase gene is essential for modifying cellular redox homeostasis under abiotic stress. In the present study, many glycosyltransferase genes were up-regulated by EBR (Table 7), which suggested that the up-regulation of the genes may confer cold tolerance in EBR-treated pepper. Cellulose synthase was also up-regulated, providing primary interface for plant environment interactions, and was associated the formation of cell wall for performing sophisticated strategies to respond to different environmental stresses.

**Figure 7:**
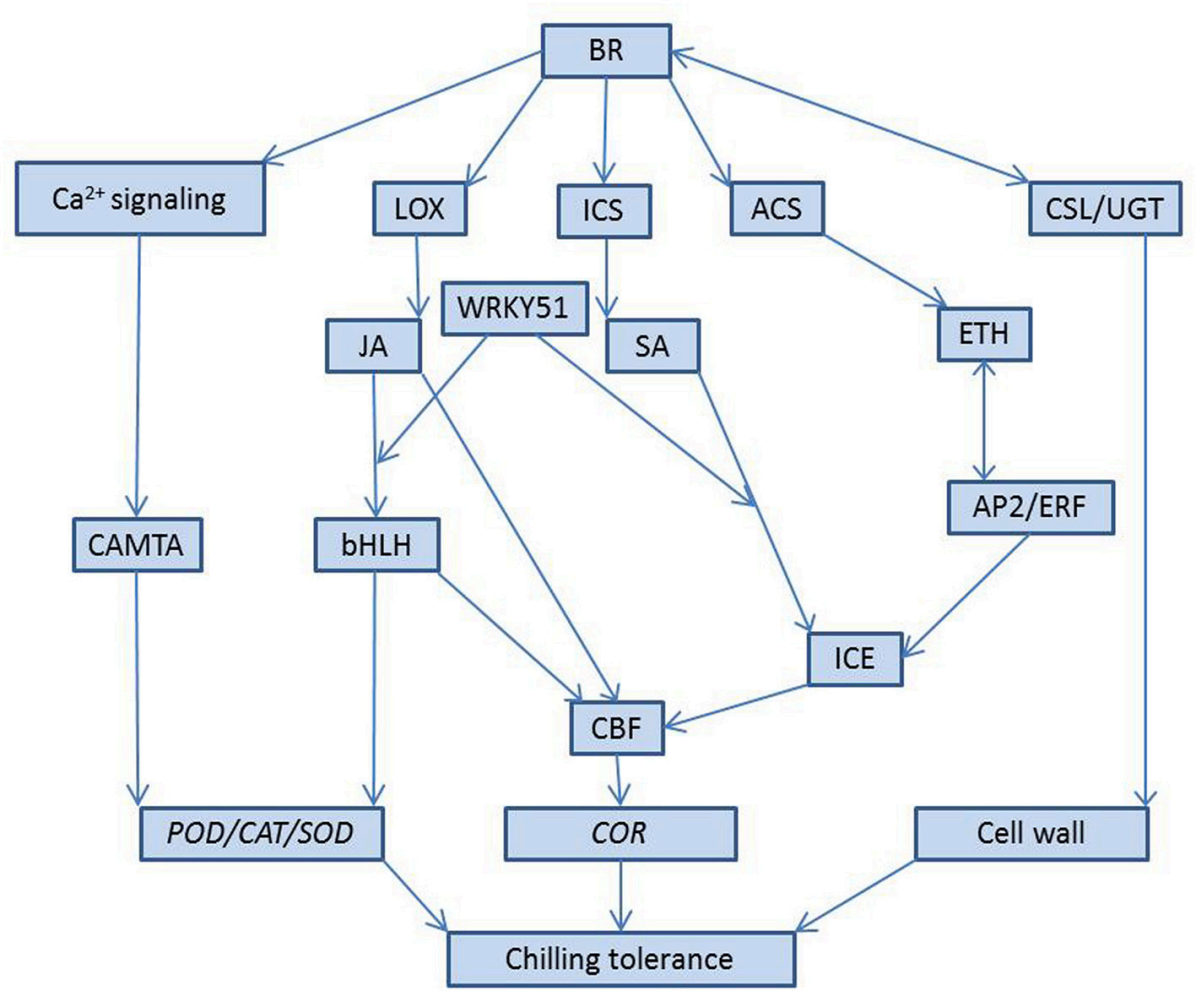
**Model depicting BR-mediated chilling tolerance via Ca^2+^, JA, SA, ETH signaling**. *LOX, ICS*, and *ACS* are jasmonic acid biosynthesis gene, salicylic acid-related gene isochorismate synthase, and ethylene biosynthesis genes ACC synthase, respectively. WRKY51, bHLH, and AP2/ERF are all transcriptional facts. CSL is cellulose synthase-like protein; UGT is UDP-glycosyltransferase. Cellular redox homeostasis related genes: *GSTX1, PER72*, and *CAT2*. CAMTA: CaM-binding transcription activator.

Taken together, the BR-induced cold stress signals are perceived by several receptors at the cell membrane, followed by calcium and hormones molecules transduction to activate downstream stress-responsive genes in response to chilling stress. Among these, BR-induced signaling pathways might regulate transcription or directly/indirectly interact with several other signaling networks. BR promoted the change of Ca^2+^ in the cytoplasm- and induced a series of biochemical reactions to regulate cellular redox homeostasis related genes, such as *GSTX1, PER72*, and *CAT2*. The key transcriptional factor bHLH of the JA signaling pathway interacted with MaICE1, and then activated the expression of downstream CBF related genes in response to chilling stress. JA, as the key upstream signal of ICE-CBF/DREB1 pathways, positively regulated cold-related gene expression in EBR-treated pepper and regulated *GSTX1, PER72*, and *CAT2* expression. We also found that BR induced SA signaling for antistress effectsby positively regulating the expression of the CBF upstream gene *ICE* and that *WRKY51* may be a potential point of cross-talking among JA, SA, and BR, where BR induces a subset of SA- or JA-responsive genes (Figure 7). BR negatively regulated the ETH signaling components and the TFs *AP2*/*ERF*, which indicated that BR induces the expression of cold related genes and the expression depends on the ETH signaling pathway. In addition, BR activated cellulose synthase-like protein (*CSLE6* and *CSLH1*), which regulated the formation of a cell wall, and BR also activated UDP-glycosyltransferase genes associated with hormone metabolism, such BR glucoside, and other metabolic enzymes (e.g., *PERK10, HSP*, and *LEA29*).

## Conclusion

In the study, we analyzed the gene expression profiles of BRs induced chilling tolerance in pepper using RNA-seq analysis. Our results showed that EBR induced 656 differently expressed genes, including 335 up-regulated and 321 down-regulated DEGs. Using GO and KEGG pathway analysis, we found that EBR application under chilling stress positively regulated photosynthesis-related genes, cellulose synthase-like protein, UDP-glycosyltransferase, and cellular redox homeostasis-related genes (*GSTX1, PER72*, and *CAT2*). Moreover, we present a model to explain the possible cross-talk of BR with SA, ETH, and JA signaling pathways under BR-induced cold tolerance. Our study provides the first evidence of the potent roles of exogenous EBR at the transcriptional level, and the response to chilling stress in pepper involved the activation of extensive transcriptional activities, signaling transduction, and modulation of metabolic homeostasis.

## Author contributions

JK and JX conceived and designed the experiments. Jie L and PY performed the experiments; Jie L, ZF, and Jia L analyzed the data. GZ and JY contributed reagents/materials/analysis tools. YG and AC helped perform the analysis with constructive discussions and language polished. JX and YG approved the final version. All authors have read and approved the final manuscript.

### Conflict of interest statement

The authors declare that the research was conducted in the absence of any commercial or financial relationships that could be construed as a potential conflict of interest.

## Supplementary material

Table S1 **Gene primers used for qPCR validation in this study**.

Table S2 **Genes information**.

Table S3 **RPKM of all genes**.

Table S4 **Differential expressed genes in chilling stress with EBR-treated plant compared to only chilling treated plant**.

Table S5 **Gene ontology analyses of differential expressed genes regulated by the EBR + chilling and only chilling stress**.

Table S6 **KEGG pathway enrichment of differential expressed genes regulated by the EBR + chilling and only chilling stress**.
